# Novel Magnet and Thermoresponsive Chemosensory Electrospinning Fluorescent Nanofibers and Their Sensing Capability for Metal Ions

**DOI:** 10.3390/polym9040136

**Published:** 2017-04-10

**Authors:** Fang-Cheng Liang, Yi-Ling Luo, Chi-Ching Kuo, Bo-Yu Chen, Chia-Jung Cho, Fan-Jie Lin, Yang-Yen Yu, Redouane Borsali

**Affiliations:** 1 Institute of Organic and Polymeric Materials, National Taipei University of Technology, Taipei 10608, Taiwan; frank62112003@yahoo.com.tw (F.-C.L.); L80860@yahoo.com.tw (Y.-L.L.); leodapon@gmail.com (B.-Y.C.); ppaul288@yahoo.com.tw (C.-J.C.); joeylin0430@gmail.com (F.-J.L.); 2 Institute of Glycosciences, Grenoble Alpes University, CNRS, CERMAV UPR 5301, 38000 Grenoble, France; borsali@cermav.cnrs.fr; 3 Department of Materials Engineering, Ming Chi University of Technology, New Taipei 24301, Taiwan; yyyu@mail.mcut.edu.tw; 4 Department of Chemical and Materials Engineering, Chang Gung University, Taoyuan 33302, Taiwan

**Keywords:** electrospun nanofibers, heavy metal ions, magnetic, fluorescent sensing, chemosensory

## Abstract

Novel multifunctional switchable chemosensors based on fluorescent electrospun (ES) nanofibers with sensitivity toward magnetism, temperature, and mercury ions (Hg^2+^) were prepared using blends of poly(*N*-isopropylacrylamide)-*co*-(*N*-methylolacrylamide)-*co*-(Acrylic acid), the fluorescent probe 1-benzoyl-3-[2-(2-allyl-1,3-dioxo-2,3-dihydro-1Hbenzo[de]isoquinolin-6-ylamino)-ethyl]-thiourea (BNPTU), and magnetite nanoparticles (NPs), and a single-capillary spinneret. The moieties of *N*-isopropylacrylamide, *N*-methylolacrylamide, acrylic acid, BNPTU, and Iron oxide (Fe_3_O_4_) NPs were designed to provide thermoresponsiveness, chemical cross-linking, Fe_3_O_4_ NPs dispersion, Hg^2+^ sensing, and magnetism, respectively. The prepared nanofibers exhibited ultrasensitivity to Hg^2+^ (as low as 10^−3^ M) because of an 80-nm blueshift of the emission maximum (from green to blue) and 1.6-fold enhancement of the emission intensity, as well as substantial volume (or hydrophilic to hydrophobic) changes between 30 and 60 °C, attributed to the low critical solution temperature of the thermoresponsive *N*-isopropylacrylamide moiety. Such temperature-dependent variations in the presence of Hg^2+^ engendered distinct on–off switching of photoluminescence. The magnetic ES nanofibers can be collected using a magnet rather than being extracted through alternative methods. The results indicate that the prepared multifunctional fluorescent ES nanofibrous membranes can be used as naked eye sensors and have the potential for application in multifunctional environmental sensing devices for detecting metal ions, temperature, and magnetism as well as for water purification sensing filters.

## 1. Introduction

In recent years, identifying the presence and concentration of heavy metal ions has received gradually increasing levels of attention because of emerging environmental and human health issues [1,2]. Heavy transition metal (HTM) cations and their derivatives are used extensively in the industrial sector, and can cause adverse environment and health problems [3,4]; thus, developing sensitive and selective fluorescent chromogenic probes composed of chelating ligands that are used to detect HTM cations in biological and environmental sensory devices is crucial. Colorimetric derivative probes based on 1,8-naphthalimide have been used extensively in fluorescence detection for HTMs such as Hg^2+^, Cd^2+^, and Cu^2+^ because of their many excellent optical properties such as high quantum yields and high photo stability [5,6,7,8,9,10,11]. A new fluorescent chemodosimeter, namely a 1,8-naphthalimide-based colorimetric derivative, 1-benzoyl-3-[2-(2-allyl-1,3-dioxo-2,3-dihydro-1Hbenzo[de]isoquinolin-6-ylamino)-ethyl]-thiourea (BNPTU), with high selectivity for Hg^2+^ in aqueous solutions and based on the reactivity of thiourea derivatives toward Hg^2+^ was reported by Liu et al. [5]. Mercury-triggered intramolecular cyclization of thiourea results in the formation of a highly blue fluorescent naphthalimide derivative, whereas the dosimeter itself fluoresces yellowish green.

Poly(*N*-isopropyl acrylamide) (PNIPAM) is a well-known thermosensitive polymer that exhibits a low critical solution temperature (LCST) of 32 °C, close to the healthy human body temperature. PNIPAM exhibits a hydrophilic extended structure below its LCST, whereas above its LCST, it dehydrates and exhibits a compact structure [12]. Chen reported that introducing hydrophilic acrylic acid (AA) monomers with different compositions can change the thermal responses of PNIPAM microgels. Furthermore, the AA content of poly(NIPAM-*co*-AA) can be adjusted to change the LCST, with the hydrogen bonding interaction being the fundamental cause of the change [13]. However, all of the aforereferenced studies adopted solutions and polymer composites rather than nanofibers in sensory applications. The high surface-to-volume ratio of nanofibers could induce responses in metal ions-sensitive, temperature-sensitive, and multifunctional sensory materials.

Electrospinning is an easy, versatile, and inexpensive technique that enables the assembly of various functional nanofibers [14,15,16,17]. The high surface-to-volume ratio of electrospun (ES) nanofibers has encouraged extensive research on sensory applications including sensors for pH levels [18], temperatures [19,20], nitric oxide [21], and metal ions [22,23,24,25,26]. Recently, various fluorescent sensor-based ES polymer nanofibers for sensing pH or different metal ions such as Hg^2+^, Fe^3+^, Zn^2+^, and Cu^2+^ were successfully prepared by our research group [24,25,26]. These multifunctional fluorescent ES nanofibers exhibited distinct on–off switching capabilities such as quenching–enhancing photoluminescence (PL) intensity and changed–recovered PL colors for sensing various pH levels or metal ions.

Poly(acrylic acid) (PAA) is a weak polyelectrolyte often used to change the surface properties of inorganic particles, because of the strong chelation of its numerous carboxyl groups with metal ions. Iron oxide (Fe_3_O_4_) nanoparticles (NPs) obtained through a one-pot method with PAA can solve the agglomeration problem [27,28,29,30,31]. Recently, Liu reported the fabrication of Fe_3_O_4_ NP, PAA, and polyvinyl alcohol composite ES nanofibers with a homogeneous dispersion of Fe_3_O_4_ NPs and high water resistance [32]. In addition, Xu et al. developed a material for industrial wastewater treatment combining polydopamine-coated mesh with PAA. This material not only selectively detects Hg^2+^ but also effectively separates oil and water, a process attributed to the hydrophilicity of PAA and the change in mesh wettability based on the chelation between Hg^2+^ and PAA [33]. However, most studies on polymer-inorganic composites have focused on exploring their physical properties, which have seldom been studied in terms of their combination with metal ions, temperature, and magnetic mulfunctional materials in sensory applications. This paper reports the production of novel multifunctional switchable ES nanofiber sensors from blends of multifunctional copolymers and Fe_3_O_4_ NPs. Comprising traditional polymer-inorganic composite ES nanofibers, our chemosensors can simultaneously detect metal ions, temperature, and magnetism.

**Figure 1:**
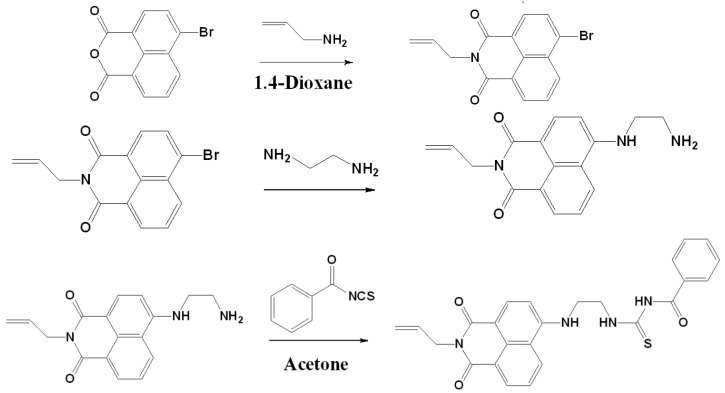
Synthesis of BNPTU fluorescent monomer.

**Figure 2:**
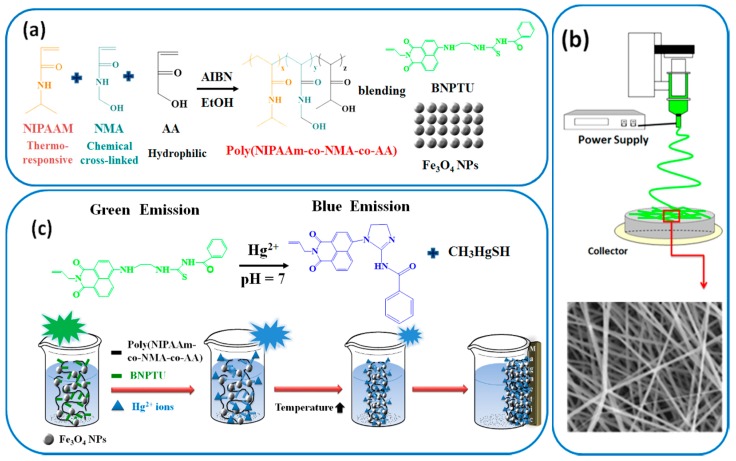
Design of multifunctional sensory electro spun (ES) nanofibers synthesized from poly(NIPAAm-*co*-NMA-*co*-AA), BNPTU, and Fe_3_O_4_ blends with magnetic fluorescence emission. (**a**) Polymerization and chemical structure of poly(NIPAAm-*co*-NMA-*co*-AA), BNPTU, and Fe_3_O_4_ particles. (**b**) Fabrication of ES nanofibers from the blends. (**c**) Change in the chemical structure of BNPTU in solutions containing Hg^2+^. The fluorescence emission from the ES nanofibers exhibited color changes. A magnet can directly attract the ES nanofibers because of the magnetism of Fe_3_O_4_ NPs.

In this study, we developed magnetic fluorescent sensory ES nanofibers by using blends of poly(*N*-isopropylacrylamide)-*co*-(*N*-methylolacrylamide)-*co*-(acrylic acid) (poly(NIPAAm-*co*-NMA-*co*-AA)), fluorescent BNPTU, and Fe_3_O_4_ NPs and a single-capillary spinneret. The nanofibers, which can sense Hg^2+^, temperature, and magnetism simultaneously, were prepared by combining synthesis and electrospinning, and their optical applications and morphological characteristics were analyzed. The process for synthesizing fluorescent BNPTU probes is shown in Figure 1. The poly(NIPAAm-*co*-NMA-*co*-AA) was synthesized through free radical polymerization (Figure 2a). The moieties of *N*-isopropylacrylamide (NIPAAm), *N*-methylolacrylamide (NMA), AA, BNPTU, and Fe_3_O_4_ NPs were designed to achieve thermoresponsiveness, chemical cross-linking, hydrophilicity, Hg^2+^ sensing, and magnetism, respectively. A single-capillary spinneret was used to fabricate ES nanofibers from poly(NIPAAm-*co*-NMA-*co*-AA) blended with BNPTU (10%) and Fe_3_O_4_ NPs (5%). The nanofibers were then post-treated through chemical cross-linking to enhance their stability in water (Figure 2b). The fluorescence emission of BNPTU is highly selective and Hg^2+^ dependent. When BNPTU is used to detect Hg^2+^, its fluorescence emission changes from green to blue, and the NIPAAm and Fe_3_O_4_ NPs exhibit thermoresponsive magnetic properties, respectively (Figure 2c). The relationship between the morphology of the ES nanofibers and their photophysical properties together with their sensing behavior in aqueous solutions was systematically investigated. The favorable detection of Hg^2+^, temperature, and magnetism demonstrated by the experimental results suggests that ES nanofibrous membranes can be used as naked eye sensors, and have the potential for application in multifunctional environmental sensing devices.

## 2. Experimental Section

### 2.1. Materials

*N*-Isopropylacrylamide (NIPAAm) and *N*-methylolacrylamide (NMA) were provided by Tokyo Chemical Industry Co. Japan. NIPAAm was re-crystallized three times from hexane–toluene (10/1, *v*/*v*) prior to use. Acrylic acid (AA, Aldrich, Saint Louis, MO, USA, 99%) was dried over CaH_2_ to distill in a round-bottomed flask overnight, then purified by passing it through a short aluminum-oxide column (50–200 μm), and stored at 4 °C prior to use. The radical initiator 2,2′-azobis(2-methylpropionitrile) (AIBN) was purchased from UniRegion Bio-Tech and was recrystallized twice in ethanol prior to use. Iron (II) (FeCl_2_∙4H_2_O) and Iron (III) (FeCl_3_∙6H_2_O) chloride hexahydrate (Aldrich, 99%), dichloromethane (Tedia, Fairfield, OH, USA, 99.9%); methanol (Tedia, HPLC/SPECTRO); NH_4_OH (Alfa, 28%); anhydrous tetrahydrofuran (99.9%); acetone (99.8%); calcium hydride (reagent grade 95%); diethyl ether (99%); 4-bromo-1,8-naphthalic anhydride (95%); benzoyl isothiocyanate (98%); allylamine (98%); 1,2-ethylenediamine (99%); chloroform (anhydrous, 99%); *N*,*N*-dimethylformamide (99%); 1,4-dioxane (99.5%); acetonitrile (99%); ethanol (99.8%); and hydrazine hydrate (reagent grade, N_2_H_4_ 50–60%) were used as received. Perchlorate salts of metal ions (Co^2+^, Cu^2+^, Fe^2+^, Mg^2+^, Hg^2+^, Pb^2+^, Zn^2+^, Cd^2+^, Ni^2+^) were purchased from Sigma-Aldrich (Saint Louis, MO, USA).

### 2.2. Synthesis of 4-Bromo-N-allyl-1,8-naphthalimide

**Figure 3:**
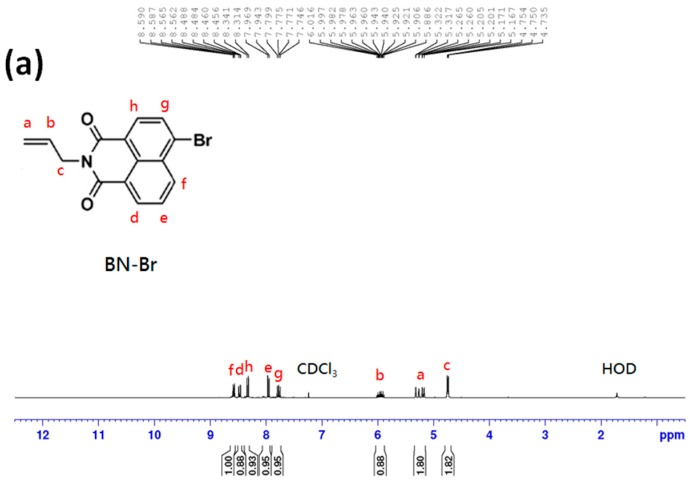

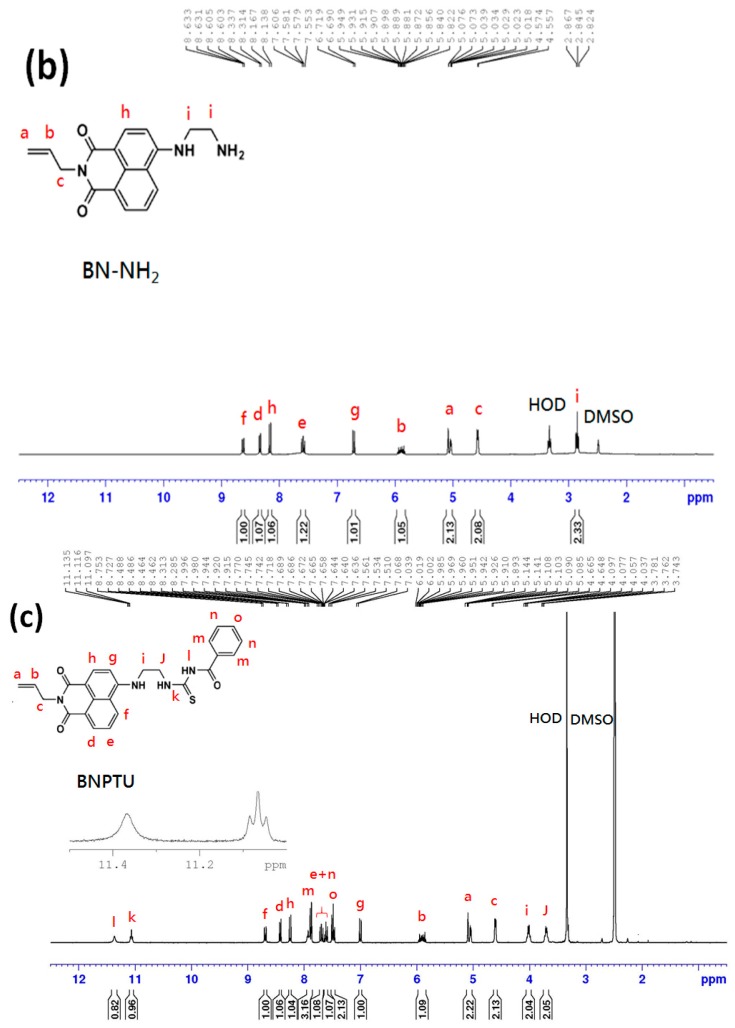
^1^H-NMR spectra recorded for (**a**) BN-Br in CDCl_3_, (**b**) BN-NH_2_ in DMSO-*d*_6_, and (**c**) BNPTU monomer in DMSO-*d*_6_.

Fluorescent BNPTU was prepared according to a previously reported method [8]. The process of its synthesis is illustrated in Figure 1. A 50-mL round-bottomed flask was charged with 4-bromo-1,8-naphthalic anhydride (1.385 g, 5 mmol), allylamine (0.317 g, 5.18 mmol), and 20 mL of 1,4-dioxane. The reaction mixture was stirred at reflux for 8 h. After cooling to room temperature, the suspension was poured into 600 mL of ice water and then filtrated. After drying in a vacuum oven overnight at room temperature, 4-bromo-*N*-allyl-1,8-naphthalimide (BN-Br) was obtained as a slightly gray solid (1.12 g, yield: 85%). ^1^H-NMR (300 MHz, CDCl_3_, TMS, Figure 3a): δ = 5.33 (a, 2H, –C*H*_2_CHCH_2_–); δ = 5.91 (b, 1H, –CH_2_C*H*CH_2_–); δ = 4.58 (c, 2H, –CH_2_CHC*H*_2_–); δ = 8.56 (d, 1H, 7–C*H*); δ = 8.03 (e, 1H, 3–C*H*); δ = 8.64 (f, 1H, 5–C*H*); δ = 7.94 (g, 1H, 6–C*H* ); δ = 8.45 (h, 1H, 2–C*H*).

### 2.3. Synthesis of 4-(Aminoethylene)amino-N-allyl-1,8-naphthalimide

A 25-mL round-bottomed flask was charged with NP-Br (500 mg, 1.57 mmol) and an excess of 1,2-ethylenediamine (8 mL). The reaction mixture was stirred at reflux for 6 h. The residues were dissolved in CH_2_Cl_2_ (200 mL) and extracted with water. Finally, 4-(aminoethylene) amino-*N*-allyl-1,8-naphthalimide (NP-NH_2_) was obtained as an orange crystal (400 mg, yield: 80%). ^1^H-NMR (300 MHz, DMSO-*d*_6_, TMS, Figure 3b): δ = 5.33 (a, 2H, –C*H*_2_CHCH_2_–); δ = 5.91 (b, 1H, –CH_2_C*H*CH_2_–); δ = 4.58 (c, 2H, –CH_2_CHC*H*_2_–); δ = 8.56 (d, 1H, 7–C*H*); δ = 8.03 (e, 1H, 3–C*H*); δ = 8.64 (f, 1H, 5–C*H*); δ = 7.94 (g, 1H, 6–C*H*); δ = 8.45 (h, 1H, 2–C*H*); δ = 2.85 (i, 2H, –C*H*_2_NH_2_).

### 2.4. Synthesis of 1-Benzoyl-3-[2-(2-allyl-1,3-dioxo-2,3-dihydro-1Hbenzo[de]isoquinolin-6-ylamino)-ethyl]-thiourea (BNPTU)

A 25-mL round-bottomed flask was charged with NP-NH_2_ (400 mg, 1.36 mmol), benzoyl isothiocyanate (0.22 g, 1.36 mmol), and 12 mL of acetone. The reaction mixture was stirred at reflux for 1 h. After cooling to room temperature, the solution was filtrated and washed with ethanol. The product was purified using chromatography with CH_2_Cl_2_ (320 mg, yield: 80%). ^1^H-NMR (300 MHz, DMSO-*d*_6_, TMS, Figure 3c): δ = 5.33 (a, 2H, –C*H*_2_CHCH_2_–); δ = 5.91 (b, 1H, –CH_2_C*H*CH_2_–); δ = 4.58 (c, 2H, –CH_2_CHC*H*_2_–); δ = 8.41 (d, 1H, 7–C*H*); δ = 7.62–7.88 (e + n, 3H, 6–C*H*; –COCCHC*H*CHC*H*–); δ = 8.72 (f, 1H, 5–C*H*); δ = 7.03 (g, 1H, 3–C*H*); δ = 8.26 (h, 1H, 2–C*H*); δ = 4.02 (i, 2H, –NC*H*_2_CH_2_N–); δ = 3.95 (j, 2H, –NCH_2_C*H*_2_N–); δ = 11.08 (k, 1H, –CSN*H*–); δ = 11.38 (l, 1H, –CON*H*–); δ = 7.48 (m, 1H, –COCCHC*H*–); δ = 7.93 (o, 2H, –COCC*H*–; –COC(CH)_4_C*H*–).

### 2.5. Synthesis of Magnetic Iron Oxide (Fe_3_O_4_) Nanoparticles (NPs)

Magnetic nanoparticles, Fe_3_O_4_, were prepared by a coprecipitation method as reported [27]. A 100-mL round-bottomed flask was charged with FeCl_3_∙6H_2_O (380 mg, 1.4 mmol), FeCl_2_∙4H_2_O (140 mg, 0.7 mmol) and 80 mL of water. The reaction mixture was stirred at reflux under N_2_ until the mixture had completely dissolved. The ammonium hydroxide (8 mL) was added dropwise under mechanical stirring at 60 °C for 30 min under N_2_. Then, the magnetic iron oxides were isolated from the solution by a magnet bar and dried in a vacuum oven at 40 °C for 24 h.

### 2.6. Synthesis of Poly(NIPAAm-co-NMA-co-AA)

Poly(NIPAAm-*co*-NMA-*co*-AA) was synthesized through free-radical copolymerization of the following three monomers: NIPAAm, NMA, and AA (Figure 2) Poly(NIPAAm-*co*-NMA-*co*-AA) with different monomer ratios were denoted **P1**–**P2**, as listed in Table 1. The concentration of AIBN used as the initiator was 0.004 M. The reaction mixture, containing dimethylformamide (DMF) and monomers, was degassed by first bubbling nitrogen through for 30 min and then left to react at 70 °C for 24 h. The reaction mixture was quenched by exposure to air. The mixture was diluted with methanol to remove the unreacted monomers. The light white filtrate was concentrated, re-precipitated from diethyl ether, collected by filtration, and dried under vacuum to obtain the polymer product. The synthesis and characterization of **P1**–**P2** are described in the following sections. The number-averaged molecular weight (*M*_n_) and polydispersity index (PDI) estimated from gel permeation chromatography (GPC) (THF eluent) are listed in Table 1 and Figure S1.

### 2.7. Synthesis of Poly(NIPAAm-co-NMA) Random Copolymers *(**P1**)*

**Figure 4:**
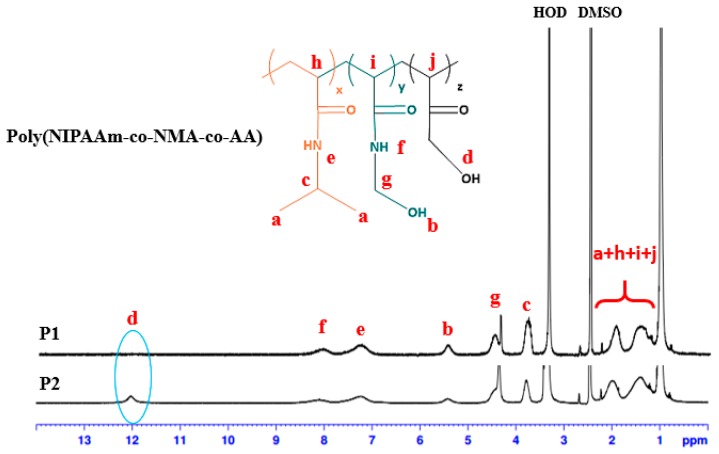
^1^H-NMR spectrum of poly(NIPAAm-*co*-NMA-*co*-AA) in DMSO.

A reaction mixture of 1.131 g (9.99 mmol) of NIPAAm, 505 mg (4.99 mmol) of NMA, 5 mg (0.03 mmol) of AIBN, and 7.5 mL of EtOH was used to produce an orange solid (yield: 86%). Figure 4 and Table 1 present the molecular weight and chemical structure characterization of poly(NIPAAm-*co*-NMA) obtained using GPC with THF as the eluent and ^1^H-NMR, respectively. The copolymer composition, estimated by performing peak integration, was consistent with the proposed structure. The estimated copolymer ratio of poly(NIPAAm-*co*-NMA) based on the NMR spectrum was 81:19, and the number-averaged molecular weight *M*_n_ and PDI estimated using GPC were 26,856 and 2.01, respectively. ^1^H-NMR (300 MHz, DMSO-*d*_6_, TMS, Figure 4): δ = 0.82–1.12 (a, 6H, –CH(C*H*_3_)_2_); 1.23–1.46 (h + i, 4H, –*CH*_2_CH–, –C*H*_2_CH–); 1.84–2.10 (h + i, 2H, –*C*H_2_C*H*–, –CH_2_C*H*–,); 5.34–5.53 (b, 1H, –NH*C*H_2_O*H*); 3.71–3.82 (c, 1H,–C*H*(*C*H_3_)_2_); 7.23–7.59 (e, 1H, –CO*NH*CH–); 7.92–8.14 (f, 1H, –*NH*CH_2_OH); 4.36–4.65 (g, 2H, –NH*CH*_2_OH–).

### 2.8. Synthesis of Poly(NIPAAm-co-NMA-co-AA) Random Copolymers *(**P2**)*

A reaction mixture of 1.131 g (9.99 mmol) of NIPAAm, 505 mg (4.99 mmol) of NMA, 0.204 mL (0.0029 mmol) of AA, 5 mg (0.03 mmol) of AIBN, and 9 mL of EtOH was used to produce an orange solid (yield: 82%). Figure 4 and Table 1 present the molecular weight and chemical structure characterization of poly(NIPAAm-*co*-NMA-*co*-AA) obtained using GPC with THF as the eluent and ^1^H NMR, respectively. The copolymer composition, estimated by performing peak integration, was consistent with the proposed structure. The copolymer ratio of poly(NIPAAm-*co*-NMA-*co*-AA) based on the NMR spectrum was 72:15:13, and the number-averaged molecular weight *M*_n_ and PDI estimated using GPC were 18041 and 1.76, respectively. ^1^H-NMR (300 MHz, DMSO-*d*_6_, TMS, Figure 4): δ = 0.82–1.12 (a, 6H, –CH(C*H*_3_)_2_); 1.23–1.46 (h + i + j, 6H, –*CH*_2_CH–, –C*H*_2_CH–, –C*H*_2_CH–); 1.84–2.10 (h + i + j, 3H, –*C*H_2_C*H*–,–CH_2_C*H*–, –CH_2_C*H*–); 5.34–5.53 (b, 1H, –NH*C*H_2_O*H*); 3.71–3.82 (c, 1H, –C*H*(*C*H_3_)_2_); 11.92–12.05 (d, 1H, –CO*C*H_2_O*H*); 7.23–7.59 (e, 1H, –CO*NH*CH–); 7.92–8.14 (f, 1H, –*NH*CH_2_OH); 4.36–4.65 (g, 2H, –NH*CH*_2_OH–).

### 2.9. Preparation of Electrospun (ES) Nanofibers

As shown in Figure 2b, the ES nanofibers were prepared using a single-capillary spinneret in a procedure similar to that described in our previous papers [19,20,21,22,23,24,25,26]. The poly(NIPAAm-*co*-NMA) (**P1**), poly(NIPAAm-*co*-NMA-*co*-AA) (**P2**), BNPTU, and Fe_3_O_4_ NPs blend was dissolved in methanol (MeOH) as solvent at 250 mL h^−1^ and stirred overnight. The blend composition (wt %) of (**P1** or **P2)**, BNPTU, and Fe_3_O_4_ NPs for preparing the ES nanofibers was 85/10/5. The polymer solution was fed into a metallic needle using syringe pumps (Model 100, KD Scientific, Holliston, MA, USA) at a feed rate of 0.8–1.0 mL h^−1^. The tip of the needle was connected to a Chargemaster CH30P high voltage power supply (Simco, Hatfield, PA, USA) set at 13.4 kV during electrospinning. A piece of aluminum foil or quartz was placed 15 cm below the tip of the needle for 30 min to collect the ES nanofibers. All experiments were performed at room temperature and a relative humidity of approximately 30%. **P1** and **P2** blended with the Fe_3_O_4_ NPs at a 5 wt % ratio are denoted by **P1-5%** and **P2-5%**. The ES nanofibers were annealed at 100 °C for 24 h in an oven for chemical cross-linking.

### 2.10. Characterization

^1^H-NMR data were recorded at room temperature using an AM 300 (300 MHz) spectrometer (Bruker, Billerica, MA, USA) and the residual proton resonance of deuterated chloroform and deuterated dimethyl sulfoxide. High resolution electrospray ionization mass spectrometry spectra were recorded using an ion-trap time-of-flight liquid chromatograph mass spectrometer (Shimadzu, Kyoto, Japan). Gel permeation chromatography (GPC) analysis was performed using a Lab Alliance RI2000 instrument (two-column, MIXED-C and MIXED-D from Polymer Laboratories, Theale, UK) connected to a refractive index detector (Schambeck SFD GmbH, Bad Honnef, Germany). All GPC analyses were performed using a polymer and THF solution at a flow rate of 1 mL min^−1^ at 40 °C and calibrated with methyl methacrylate. The thermal decomposition temperature was determined using a Q50 thermal gravimetric analyzer (TGA) (TA Instruments, Lukens Drive, New Castle, DE, USA) over a heating range of 100–800 °C at a heating rate of 10 °C min^−1^ in a nitrogen atmosphere. The LCST of the prepared copolymer solution was recorded by monitoring the transmittance of a 520 nm light beam on Shimadzu UV–Vis spectrophotometer. The copolymer concentration in water was 1 wt %, and the temperature was raised from 10 to 70 °C in 2.5 °C increments every 10 min. A plot of the changes in optical transmittance as a function of temperature for polymers in water was made, the LCST corresponds to the first turning point of the transition curve [21]. Magnetic properties of Fe_3_O_4_ nanoparticles or ES nanofibers were measured on a Lake Shore VSM-7407 instrument (MPMS (SQUID) VSM, Quantum Design, San Diego, CA, USA). The magnetization hysteresis loops were measured at 300 K [30].

The morphologies of ES nanofibers were characterized using an S-520 scanning electron microscope (Hitachi, Tokyo, Japan) equipped with X-ray microanalysis capability. Samples were coated with platinum prior to scanning electron microscopy (SEM) characterization, and analysis was conducted at an increased voltage of 10 kV. Fluorescence optical microscopy images were captured using an LCS SP5 two-photon laser confocal microscope (Leica, Mannheim, Germany). The morphologies of the ES nanofibers were similar to those reported in our previous studies [15,20,24].

Ultraviolet–visible (UV–Vis) absorption and PL spectra were measured to examine photophysical properties and recorded using a UV–Vis spectrophotometer (Shimadzu) and a Fluorolog-3 spectrofluorometer (Horiba Jobin Yvon, Edison, NJ, USA), respectively. Variations in the optical absorption and PL of the prepared ES nanofibers with different metal ion concentrations are described as follows. To ensure that the beam excited the same point on the prepared samples during each measurement, the ES nanofibers were fixed in cuvettes with adhesive tape, and the cuvette was filled with a basic aqueous metal ion solution at 10^−4^–10^−2^ M. Each measurement was maintained for 15 min to ensure that the chelating reaction reached equilibrium. All PL spectra of the ES nanofibers were recorded using the spectrofluorometer, and the samples were excited at a suitable wavelength, as described in our previous studies [19,20,21,22,23,24,25,26].

## 3. Results and Discussion

### 3.1. Characterization of BNPTU and Poly(NIPAAm-co-NMA-co-AA)

The chemical structure of BNPTU was characterized using ^1^H-NMR (Figure 3). The synthesis routes of BNPTU (Figure 1) are similar to those reported in a previous study [8]. Figure 2a shows the route by which poly(NIPAAm-*co*-NMA-*co*-AA) copolymers were synthesized through free radical polymerization. The two types of copolymers (**P1** and **P2**) were synthesized and their composites are listed in Table 1. Figure 4 shows the ^1^H-NMR spectrum of poly(NIPAAm-*co*-NMA) (**P1**) and poly(NIPAAm-*co*-NMA-*co*-AA) (**P2**). The proton peaks at 7.23–7.59 ppm (Peak e) and 3.71–3.82 ppm (Peak c) correspond to the methylene neighbors of the secondary amine moiety and the alkyl chains on NIPAAm, respectively. The proton peaks at 5.34–5.53 ppm (Peak b), 7.92–8.14 ppm (Peak f), and 4.36–4.65 ppm (Peak g) correspond to the methylene neighbors of the hydroxyl, secondary amine moiety, and alkyl chains on NMA, respectively. The proton peak at 11.92–12.05 ppm (Peak d) corresponds to the methylene neighbor of hydroxyl on the AA in **P2**. The peak at 0.82–1.12 ppm (Peak a) and those at 1.23–2.1 ppm (Peaks h, i, and j) correspond to the alkyl chains on the copolymers. The copolymer ratios of **P1** and **P2** were estimated from NMR spectra, and were 81:19 and 72:15:13, respectively. The favorable agreement between the feeding ratio and experimental composition suggests the successful preparation of the target copolymers. The molecular weights, thermal properties, and LCSTs of **P1** and **P2** are listed in Table 1. The number-averaged molecular weights and polydispersity indices (PDIs) of **P1** and **P2** were 26,856 and 2.01, and 18,041 and 1.76, respectively. Notably, Figure S1 shows that the accurate PDI (*M*_n_/*M*_w_) data of **P1** were 26,856/53,980 equal to 2.01 and that of **P2** was 18,041/31,752 equal to 1.76. The thermal decompositon curves of the prepared polymers are presented in Figure S2 (Supplementary Materials). The identical thermal decomposition temperature of 210 °C for **P1** and **P2** is attributable to their similar NIPAAm compositions. All decompostion temperatures for **P1** and **P2** were higher than 270 °C, and thus, they exhibited favorable and stable thermal properties. Figure S3 shows the typical optical transmittance (520 nm) versus temperature curves of **P1** and **P2**, which exhibit thermoresponsive soluble-to-insoluble phase transitions in an aqueous medium. The copolymers are soluble in water below their LCSTs. The LCSTs of **P1** and **P2** are both approximately 55 °C, slightly higher than that of PNIPAM (32 °C) because of the hydrophilic characteristics of the NMA and AA compounds, as stated in our previous study [23] and one other study [34].

**Figure 5:**
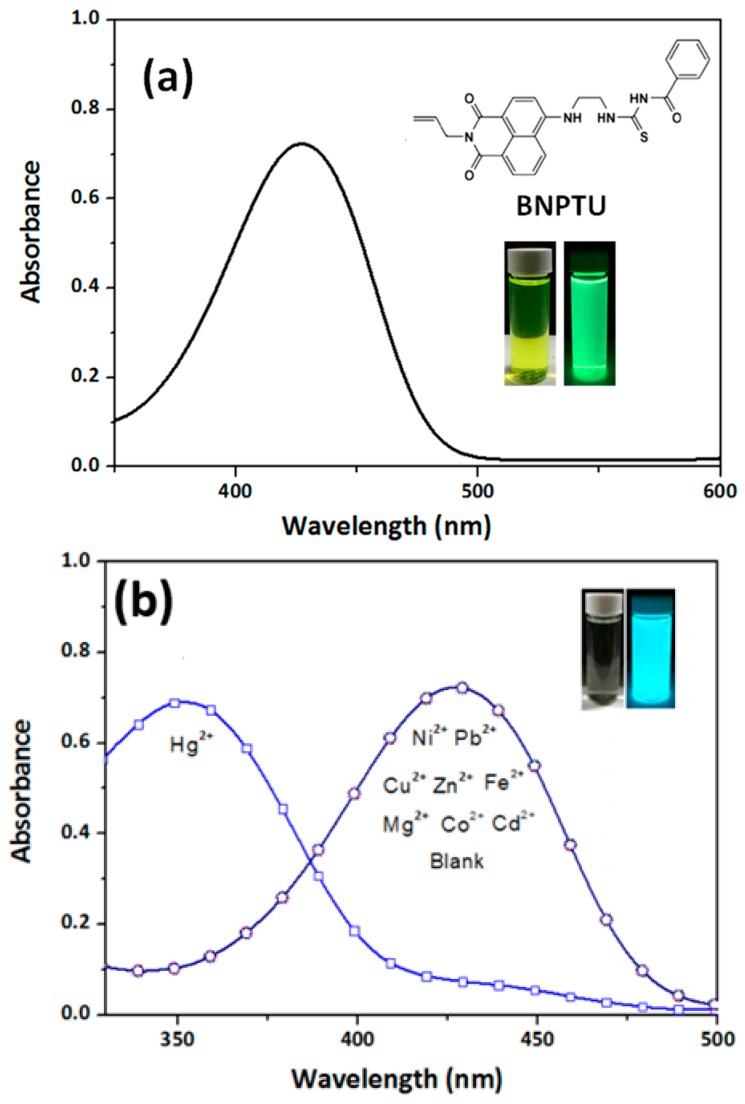
UV–Vis spectra of (**a**) BNPTU in CH_3_CN solution (10^−5^ M) and (**b**) variation of UV–Vis spectra of BNPTU CH_3_CN solution (10^−5^ M, pH 7) with different metal ions at 10^−4^ M. The corresponding inset figures show the color changes under visible light and 254-nm UV light.

Figure 5a illustrates that BNPTU in CH_3_CN had a maximum in the UV–Vis absorption peak (λ^abs^_max_) of 430 nm at pH 7 and emitted green fluorescence under UV light (inset) as it is a fluorescent dye. Figure 5b shows variations in the UV–Vis spectra of BNPTU in a CH_3_CN solution containing various metal ions at a concentration of 10^−5^ M (pH 7). The λ^abs^_max_ of BNPTU was blueshifted from 430 to 350 nm when the Hg^2+^ ion was added. The Hg^2+^ ion transformed the thiourea unit of BNPTU under aqueous conditions into an imidazoline moiety with considerably weakened electron-donating ability (Figure 2c) [5,35,36,37]. However, no change in the absorption peak was observed when other metal ions such as Co^2+^, Ni^2+^, Pb^2+^, Zn^2+^, Mg^2+^, Cu^2+^, Fe^2+^, and Cd^2+^ were added, suggesting that BNPTU exhibited high selectivity and sensitivity toward Hg^2+^.

### 3.2. Morphology and Characterization of ES Nanofibers

**Figure 6:**
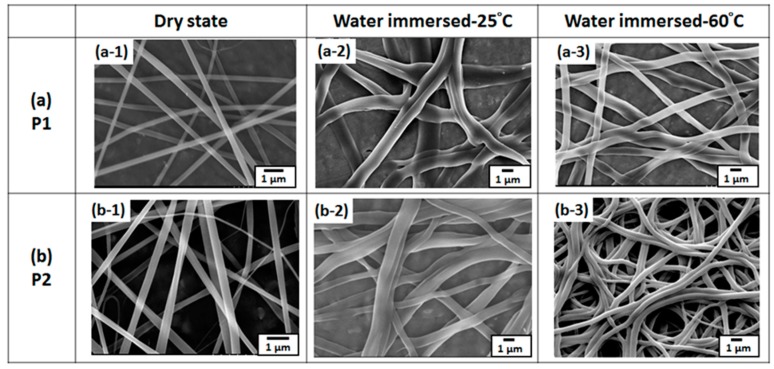
Field-emission scanning electron microscopy (FE-SEM) images of copolymers. (**a**) **P1** and (**b**) **P2** cross-linked nanofibers at 120 °C in a dry state and treated with water at 30 and 60 °C.

Figure 6 presents field-emission SEM (FE-SEM) images of the ES nanofibers prepared using **P1** and **P2** at a solution concentration of 250 mg mL^−1^ with MeOH. When dry, the **P1** and **P2** ES nanofibers had average diameters of 416 ± 31 and 437 ± 42 nm, respectively. The recorded average diameter was a statistical average of 50 fibers from each sample. Moreover, all of the ES nanofibers from pure MeOH solvent were smooth and nonporous. The diameters of the **P1** and **P2** ES nanofibers were similar when they were dry because the concentration of the ES solution was fixed and the molar ratios of the NMA and AA moieties in the copolymers were lower than that of NIPAAm. To observe the morphologies of the cross-linked **P1** and **P2** ES nanofibers in pure water at various temperatures, the fibers were collected on a small piece of aluminum foil and immersed in water at 30 °C or 60 °C. After 10 min, the samples were solidified by placing in a flask containing liquid nitrogen, and the residual water was removed using a vacuum for 30 min to retain the original morphology. Figure 6 shows the FE-SEM images of the **P1** and **P2** ES nanofibers in a wet state after they were immersed in water at 30 or 60 °C. As shown in Figure 6 (wet state), the **P1** and **P2** fibers exhibited diameters of 1250 ± 205 and 1420 ± 282 nm at 25 °C and 830 ± 124 and 920 ± 138 nm at 60 °C, respectively. The diameters were substantially enlarged after the nanofibers were immersed in water (416 ± 31, 437 ± 42 nm, respectively; Figure 6) because the hydrophilic NIPAAm chain swelled in the water. However, these swollen fibers maintained their cylindrical shape and did not dissolve in water, a phenomenon attributed to the efficient chemical cross-linking of the NMA moiety [23]. Furthermore, the fiber diameters at 30 °C were greater than those at 60 °C because of the hydrophilic NIPAAm chain, the temperature of which was below the LCST. By contrast, the nanofiber diameters decreased from approximately 1.5 μm to 1.0 μm as the temperature increased from 30 to 60 °C, due to the temperature of which was above the LCST resulting in the hydrophobic NIPAAm chain.

**Figure 7:**
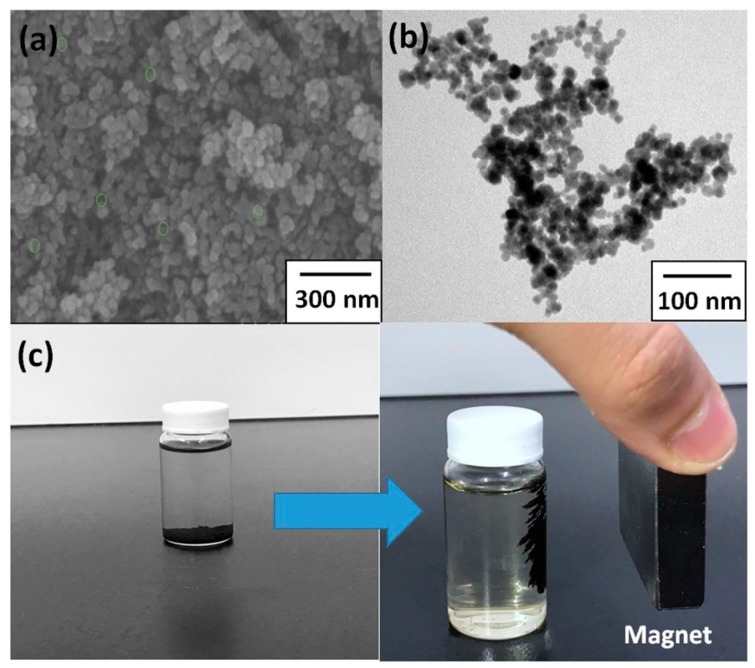
(**a**) FE-SEM and (**b**) Transmission electron microscopy (TEM) images of Fe_3_O_4_ NPs synthesized through coprecipitation. (**c**) Magnetic Fe_3_O_4_ NPs in the solution (left: Fe_3_O_4_ precipitated out of the solution and dropped to the bottom; right: Fe_3_O_4_ accumulated on the side of the bottle because of the magnet).

Figure 7a,b shows FE-SEM images and transmission electron microscopy images of the Fe_3_O_4_ NPs, respectively. The diameters of the nanoparticles were monodispersed within the range 15–25 nm. Figure 7c shows that the Fe_3_O_4_ NPs precipitated out of the solution and dropped to the bottom because of higher density (left image) and then accumulated on the side of the bottle because of a magnetic bar placed next to the bottle (right image), indicating that magnetic Fe_3_O_4_ NPs can be absorbed using a magnet [38].

**Figure 8:**
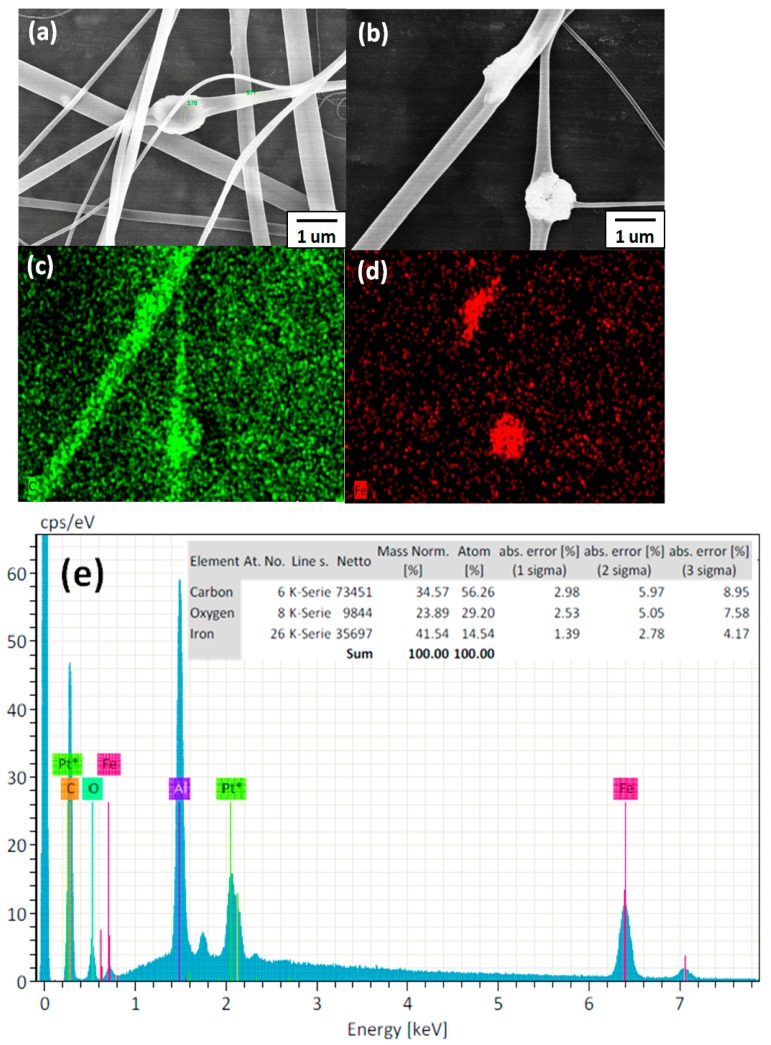
(**a**) SEM image of Fe_3_O_4_ NPs blended with nanofibers at a 5% weight ratio. (**b**) FE-SEM image of **P2-5%** cross-linked nanofibers in the presence of Fe^3+^. (**c**,**d**) Energy dispersive X-ray spectroscopy EDS maps of C and Fe within the confined area in (b). (**e**) EDS spectrum recorded within the region defined in (b).

Figure 8a presents an SEM image of **P2-5%** ES nanofibers prepared from **P2** copolymers blended with Fe_3_O_4_ NPs at a 5% weight ratio. The strong stretching force associated with electrospinning induces orientation of these Fe_3_O_4_ NPs along the axis of the fiber. The numerous carboxyl groups of PAA in **P2** inhibited the aggregation of Fe_3_O_4_ NPs, a result similar to those of several previous studies [27,28,29,30,31]. The result can be attributed to **P2-5%** containing carboxyl groups of PAA that interact with the Fe_3_O_4_ NPs, thereby enabling the PAA to be easily adsorbed by Fe_3_O_4_ NPs. Figure 8b shows an SEM image of **P2-5%** ES nanofibers before elemental mapping, and Figure 8c,d shows the corresponding SEM images of **P2-5%** ES nanofibers after carbon (C) and iron (Fe) elemental mapping, respectively, by using energy dispersive X-ray spectroscopy (EDS). Notably, the C and Fe are contributed by the **P2** and Fe_3_O_4_ NPs, respectively. The C and Fe mapping images in Figure 8b–d indicate that Fe_3_O_4_ NPs were present within the **P2-5%** ES nanofibers because no Fe species remained on the substrate, as shown by the dark region. The Fe content within the **P2-5%** ES nanofibers was 14.54 wt % as estimated according to the EDS spectrum (Figure 8e). Furthermore, the amounts of Fe_3_O_4_ NPs within the **P2-5%** ES nanofibers were confirmed through TGA in an oxygen atmosphere (Figure S4). Figure S4 shows the stages within the temperature range 100–750 °C, including the decomposition of poly(*N*-methylolacrylamide)(PNMA) and PAA, and the oxdiation of the Fe_3_O_4_ NPs. Weight loss initially occurred below 300 °C, and originated from either the decomposition of noncoordinated carboxyls or the evaporation of trace water. Weight loss that occurred between 300 and 450 °C was caused by the cleaving of PNIPAM chains [31]. Finally, the copolymers were degraded and Fe_3_O_4_ was completely transformed into Fe_2_O_3_ at 700 °C [30]. The calculated weight percentage of the remaining content, consisting of carbon residue and Fe_2_O_3_, was approximately 14 wt %.

### 3.3. Hg^2+^ Sensing, Thermoresponsiveness, and Magnetic Properties of ES Nanofibers

**Figure 9:**
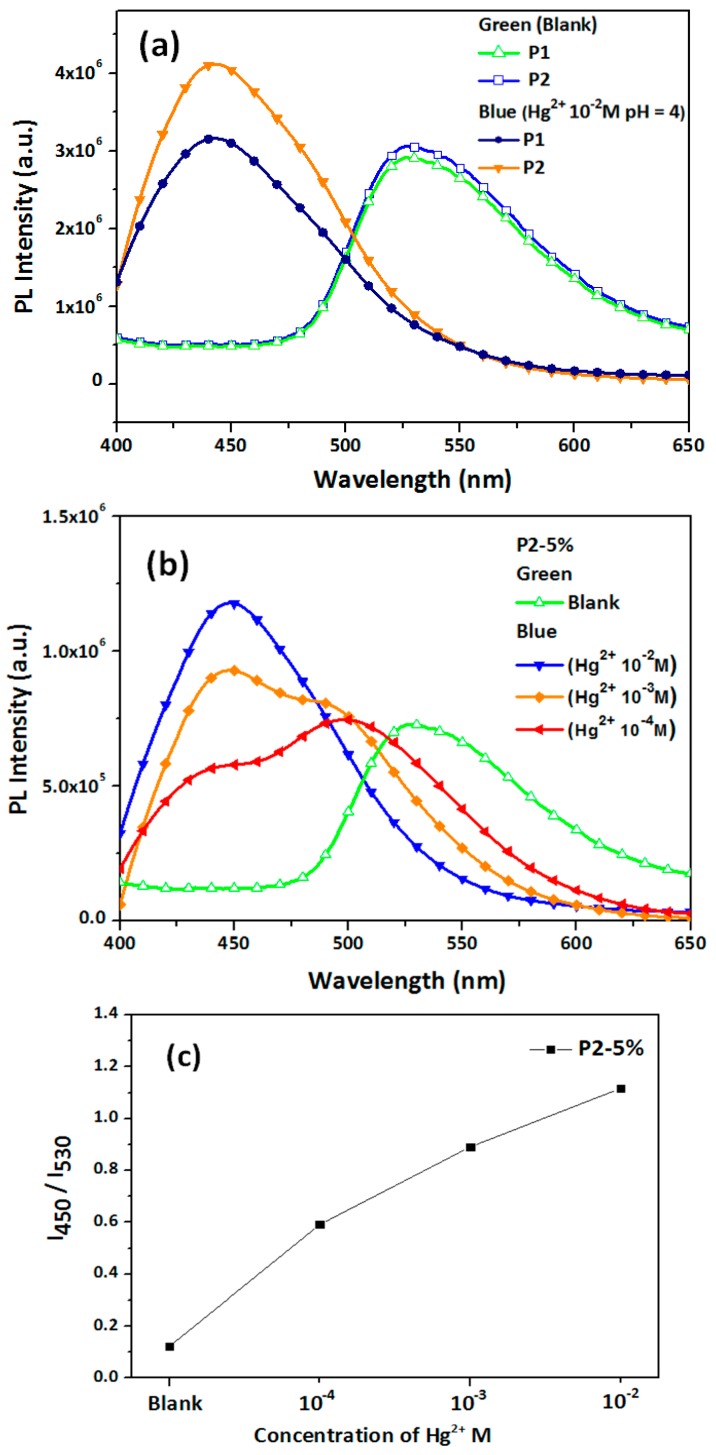

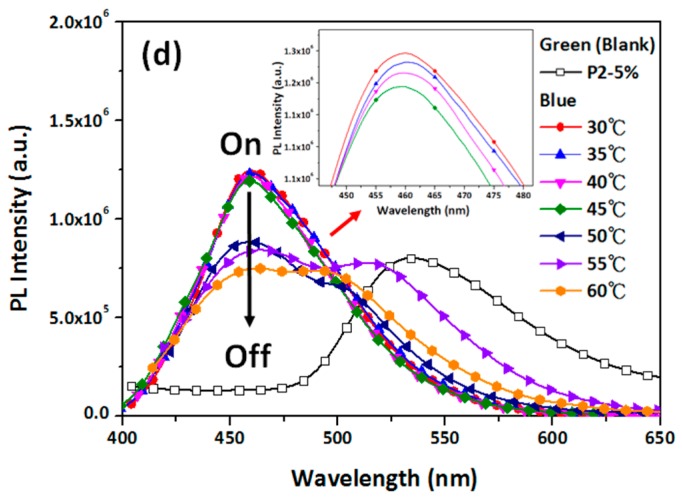
(**a**) Photoluminescence (PL) spectra of **P1** and **P2** nanofibers blended with 10 wt % BNPTU. (**b**) Comparison of **P2-5%** nanofibers with different Hg^2+^ concentrations between 10^−2^, 10^−3^ and 10^−4^ M in aqueous solution. (**c**) Relative fluorescence intensity changes (*I*_450_/*I*_530_) of **P2-5%** ES nanofibers in aqueous solutions with various Hg^2+^ concentrations. (**d**) PL spectra of **P2-5%** ES nanofibers in 10^−2^ M Hg^2+^ solution with a temperature increase from 30 to 60 °C.

**P1** or **P2** blended with 10% BNPTU and 5% Fe_3_O_4_ NP ES nanofibers, which can sense metal ions because of the ability of PAA and BNPTU to adsorb Hg^2+^, was systematically explored [8,33]. Figure 9a shows the PL spectra of ES nanofibers prepared from **P1** and **P2** blended with 10% BNPTU in an aqueous solution without Hg^2+^ (blank) or with Hg^2+^ at 10^−2^ M and subjected to 430-nm excitation. The maximum emission peak (λ^PL^_max_) of the **P1** and **P2** ES nanofibers blueshifted substantially from 530 nm in a non-Hg^2+^ aqueous solution to 450 nm in an Hg^2+^ aqueous solution. This change corresponded to the thiourea unit of BNPTU, which transformed the imidazoline moiety (with the Hg^2+^ ion), thereby causing a significant reduction in electron delocalization within the fluorophore (Figure 2c) [5,35,36,37]. Moreover, the PL intensity of the **P2** ES nanofibers was 1.3 times that of **P1** ES nanofibers, indicating that the AA moiety of **P2** enhances the adsorption of mercury ions, similar to one report [33].

Figure 9b shows the PL spectra of ES nanofibers prepared from **P2** blended with 10% BNPTU and 5% Fe_3_O_4_ NPs. **P2-5%** was placed in aqueous solutions without Hg^2+^ (blank), with Hg^2+^ at 10^−4^, 10^−3^, and 10^−2^ M, and under neutral conditions at 430-nm excitation. The presence of the highest Hg^2+^ concentration (10^−2^ M) led to a λ^PL^_max_ blueshift from 530 nm to 450 nm in the emission spectra, similar to that of the **P2** ES nanofibers without Fe_3_O_4_ NPs, indicating that the metal ion sensing ability was unaffected by Fe_3_O_4_ NPs. As shown in the figure, the concentration of Hg^2+^ increasing from 0 (blank) to 10^−4^, 10^−3^, and 10^−2^ M resulted in clear blueshifts in the λ^PL^_max_ from 530 to 500, 450, and 450 nm, respectively. Furthermore, when the mercury ion concentration was at the extremely dilute level of 10^−3^ M, the emission maximum shift Δλ_max_ was observed to be as high as 80 nm, resulting in a color change easily observable by the naked eye. Moreover, the lowest detectable Hg^2+^ concentration for the ES nanofibers was 10^−4^ M, suggesting that the **P2-5%** ES nanofibers were highly sensitive to Hg^2+^ but not to general ions, even when the system contained Fe_3_O_4_ NPs.

Figure 9c shows the changes of the fluorescence intensity ratio, *I*_450_/*I*_530_ (*I*_450_ is the fluorescence intensity of BNTPU after the detection of Hg^2+^ emission at 537 nm, whereas *I*_530_ is the fluorescence intensity of BNTPU before the detection of Hg^2+^ emission at 530 nm) of the **P2-5%** ES nanofibers when subjected to Hg^2+^. As the concentration of Hg^2+^ was increased, BNTPU emission at 530 nm gradually decreased, and BNTPU-Hg^2+^ emission at 450 nm gradually increased. These changes corresponded to the fluorescence of BNTPU chelated with Hg^2+^ ions, causing the *I*_450_/*I*_530_ to increase from approximately 0.1 to 1.2 as the Hg^2+^ ion concentration increased from 0 to 10^−2^ M. The **P2-5%** ES nanofibers exhibited high sensitivity to Hg^2+^ ions between 10^−4^ and 10^−2^ M. Moreover, the titration data of Figure 9c show that the Kd was calculated as 1.58 mM [24].

Figure 9d shows fluorescence variations in the **P2-5%** ES nanofibers in a 10^−2^ M Hg^2+^ environment as the temperature was varied from 30 to 60 °C. At 30 °C, the temperature at which the ES nanofibers chelate Hg^2+^, the λ^PL^_max_ was blueshifted substantially from 530 nm (blank, green emission) to 450 nm (blue emission) in the emission spectra. When the temperature was increased from 30 to 45 °C, only a mild decrease in PL intensity at 450 nm (λ^PL^_max_) was observed, exhibiting almost no change, as shown in the enlarged inset image. However, when the temperature was increased from 45 to 50 °C, a dramatic quenching of the PL intensity of the ES nanofibers was observed. The PL intensity decreased as the temperature increased from 50 to 60 °C, reaching its lowest point at 60 °C. Notably, Figure S5 shows no changes in the PL intensity of the pristine BNPTU compound as the temperature was increased from 30 to 60 °C because the chemical stability of BNTPU is favorable at room temperature or higher temperatures (e.g., 60 °C), indicating that the quenching of the PL intensity of the ES nanofibers (Figure 9d) was not caused by the pristine BNPTU compound.

The aforementioned thermoreversible luminescence characteristics are explained as follows. As shown in Figure 6, compared with those in the dry state, the ES fibers soaked in water (wet state) underwent morphological change at 30 °C (below the LCST). At 30 °C, the hydrophilic groups in the PNIPAM interacted easily with water molecules to form intermolecular hydrogen bonds. However, the swollen ES fibers were insoluble in water because of the chemically cross-linked NMA segment. When the temperature was raised to 60 °C (above the LCST), the intermolecular hydrogen bonds between the PNIPAM and water were broken, leading to the release of water molecules from the fibers. However, Hg^2+^ was still present within the fibers. Furthermore, as the temperature was 60 °C (above the LCST), the PNIPAM was collapsed and densely packed, potentially suppressing absorption of incident light by the BNPTU moiety with Hg^2+^, resulting in a reduction of the PL intensity. Thus, the prepared ES fibers exhibited an on–off PL intensity profile with temperature variation (Figure 9d), indicating that the ES nanofibers have thermoresponsive properties.

**Figure 10:**
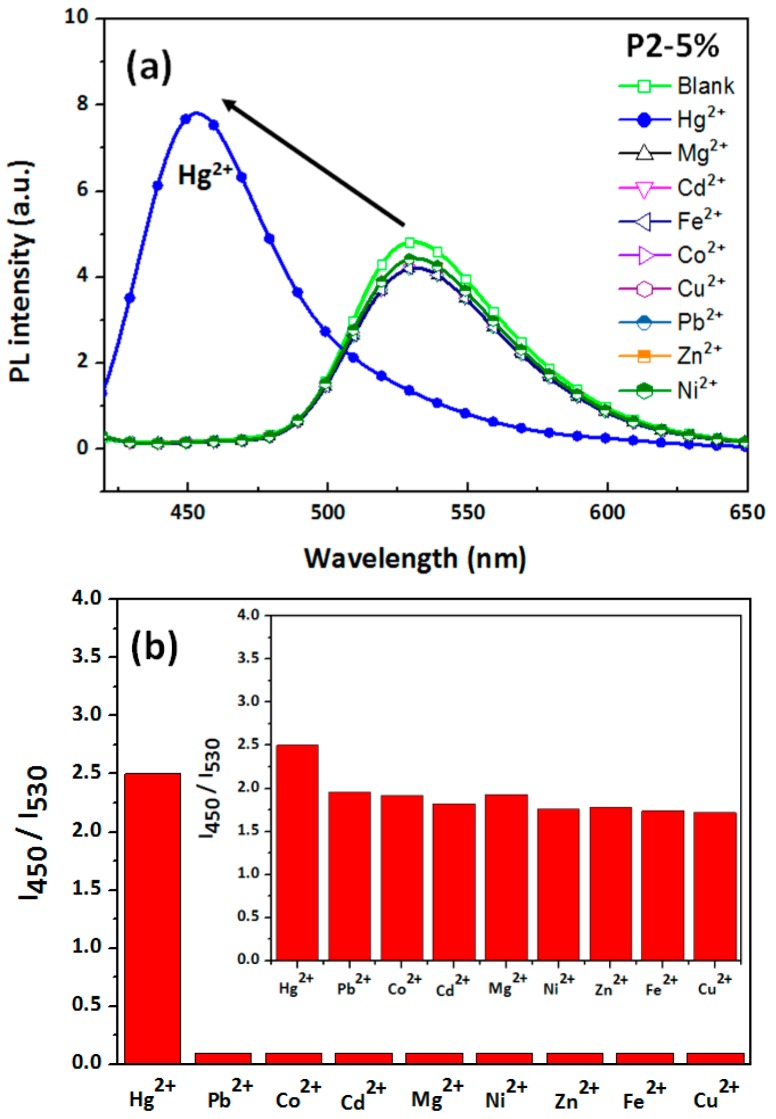
(**a**) Variation in the normalized PL spectra of **P2-5%** ES nanofibers in aqueous solutions with various metal ions (10^−2^ M) and no cation (blank). (**b**) Fluorometric responses (*I*_450_/*I*_530_) of **P2-5%** ES nanofibers to various cations at 10^−2^ M aqueous solutions. From left to right: Hg^2+^, Pb^2+^, Co^2+^, Cd^2+^, Mg^2+^, Ni^2+^, Zn^2+^, Fe^2+^, and Cu^2+^. All the inset figures show corresponding photographs recorded under UV light. Inset of (**b**): fluorimetric response of the **P2-5%** ES nanofibers to solutions containing various metal ions at 10^−2^ M (as in (**b**)) when Hg^2+^ at 10^−2^ M is present.

The selectivity of **P2-5%** ES nanofibers toward Hg^2+^ over other common metal ions was also studied (Figure 10). Figure 10a shows that among all tested metal ions, namely Hg^2+^, Pb^2+^, Co^2+^, Cd^2+^, Mg^2+^, Ni^2+^, Zn^2+^, Fe^2+^, and Cu^2+^ (10^−2^ M; all metal ion test solutions were controlled at pH 4), a substantial blueshift in PL was observed only in the presence of Hg^2+^. In Figure 8b, *I*_450_/*I*_530_ indicates the ratio of a PL intensity of 530 nm (λ^PL^_max_), corresponding to other metal ions (Hg^2+^, Pb^2+^, Co^2+^, Cd^2+^, Mg^2+^, Ni^2+^, Zn^2+^, Fe^2+^, and Cu^2+^), to a PL intensity of 450 nm (λ^PL^_max_), corresponding to Hg^2+^. The presence of Hg^2+^ induced the most prominent *I*_450_/*I*_530_ enhancement (approximately 2.5-fold), resulting in blue emission. For all of the other metal ions, the nanofibers exhibited reduced *I*_460_/*I*_510_ values (approximately 0.1-fold), resulting in green emission (Figure 10b). In addition, the fluorescence spectra recorded in the presence of Hg^2+^ ions and the other metal ions revealed that none of the other metal ions interfered with the Hg^2+^ ion-induced fluorescence enhancement (inset Figure 10b), indicating that the sensing of Hg^2+^ by the **P2-5%** ES nanofibers was virtually unaffected by commonly coexisting ions.

**Figure 11:**
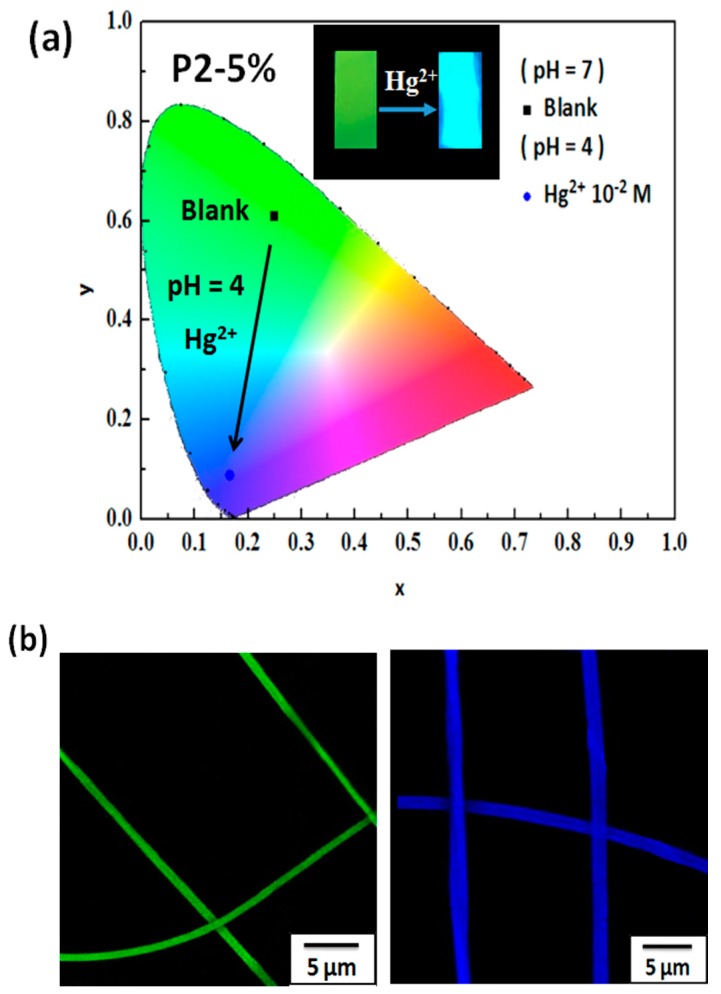

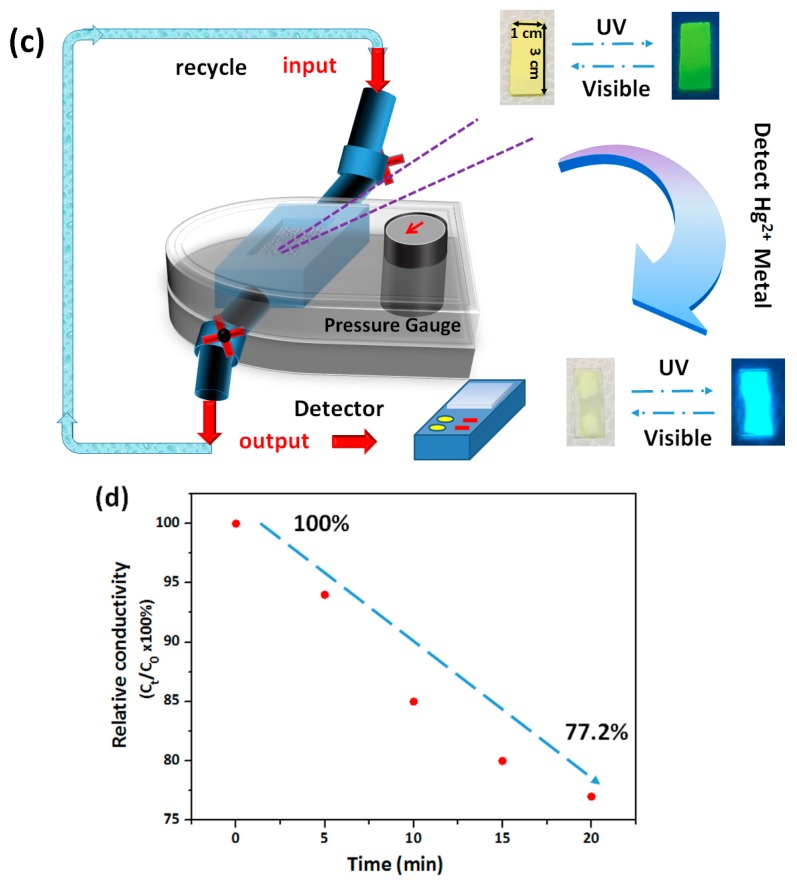
(**a**) CIE coordinates of **P2-5%** ES nanofibers in pH 7 aqueous solutions and after the detection of Hg^2+^ at 10^−2^ M aqueous solutions. (**b**) Confocal microscopy images of the ES nanofibers. All the inset figures show corresponding photographs recorded under UV light. (**c**) Schematic of a sensory filter microfluidics system for real-time metal ion sensing using an ES nanofiber membrane. (**d**) Relative conductivity versus time of the prepared Hg^2+^ solution in the microfluidics system.

Figure 11a shows the CIE coordinates of the **P2-5%** ES nanofibers in 0–10^−2^ M Hg^2+^ aqueous solutions. All of the inset figures show corresponding photographs captured under UV light. The CIE coordinates show a strong blueshift from (0.24, 0.61) (blank) to (0.16, 0.08) (pH 4, Hg^2+^ 10^−2^ M) as the concentration of Hg^2+^ was varied from 0 to 10^−2^ M because of the detection of BNPTU by Hg^2+^, resulting in a color change from green to blue in the **P2-5%** ES nanofibers. Furthermore, as shown in the confocal microscopy images in Figure 11b, the color emission of the **P2-5%** ES nanofibers varied from green to blue as the concentration of Hg^2+^ was increased. A microfluidics system was constructed, in which a **P2-5%** ES nanofiber filter membrane with an area of 3 cm^2^ placed in the middle of a tube was used to rapidly absorb and sense Hg^2+^ in a solution flowing through the tube (Figure 11c). Figure 11d depicts the measured time-dependent variation in the solution conductivity. The prepared Hg^2+^ solution contained 1 ppm Hg^2+^ (6.3 × 10^−3^ M Hg^2+^ in 0.5 L of water) and the conductivity of the Hg^2+^ solution in its initial state (0 min) was 131.2 μS cm^−1^. The solution conductivity gradually decreased to 101.4 uS cm^−1^. Table S1 contains raw data on the conductivity changes over 20 min, and Figure 11d depicts *C*_t_/*C*_0_ (%) versus time (*C*_0_: original conductivity at 0 min; *C*_t_: conductivity at time *t*). The solution conductivity decreased as the flow time increased, indicating that an increasing number of Hg^2+^ ions was absorbed by the **P2-5%** ES nanofiber membrane, yielding less Hg^2+^ in the solution. Thus, the 100% conductivity of the solution in its initial state (0 min) had decreased to 77.2% after 20 min. This rapid change in conductivity was caused by the high surface-to-volume ratio of the **P2-5%** ES nanofibers. The sensory filter membrane based on the **P2-5%** ES nanofibers specifically absorbed Hg^2+^ in an aqueous solution that contained a variety of metal ions and had a dual fluorescent chemosensory function for Hg^2+^.

**Figure 12:**
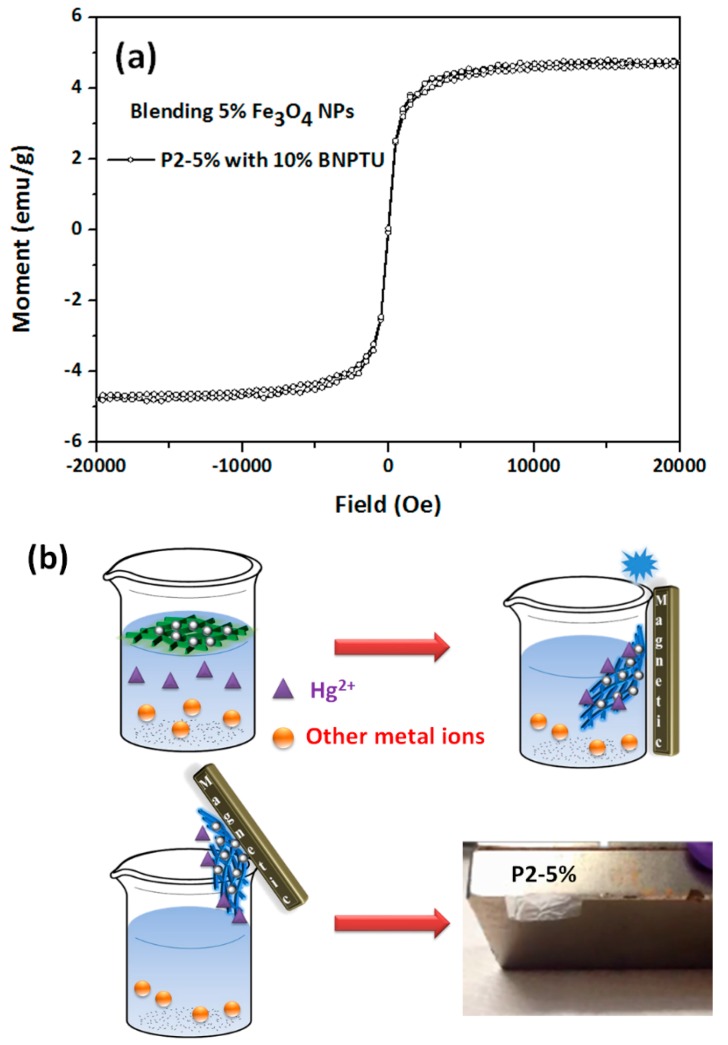
(**a**) Hysteresis loops of **P2-5%** ES nanofibers (5 wt % Fe_3_O_4_ NPs) measured at 25 °C. (**b**) Schematic of a filter sensory membrane prepared from **P2-5%** ES nanofibers composed of poly(NIPAAm-*co*-NMA-*co*-AA)), BNPTU, and Fe_3_O_4_ NPs blends to simultaneously chelate and sense Hg^2+^. A magnet can directly attract the **P2-5%** ES nanofibers because of the magnetism of Fe_3_O_4_ NPs.

Figure 12a shows the saturation magnetization and coercivity of the **P2-5%** ES nanofibers-nanocomposite, which were determined using a vibrating sample magnetometer in the field range of ±20,000 Oe at 300 K. The results indicate that the **P2-5%** ES nanofibers exhibited magnetic properties, and the saturation magnetization was 4.8 emu g^−1^. Moreover, the magnetic **P2-5%** ES nanofibers specifically adsorbed Hg^2+^ in an aqueous solution containing a variety of metal ions and had a fluorescent chemosensory response to Hg^2+^ (Figure 11). Thus, filter membranes based on the **P2-5%** ES nanofibers with porous architectures can specifically chelate with Hg^2+^ in an aqueous solution containing many types of metal ion and serve as fluorescent chemosensors for Hg^2+^. These results could assist researchers in cleaning water while simultaneously chelating and sensing Hg^2+^. In addition, rather than removal through alternative methods, a magnet can directly attract the **P2-5%** ES nanofibers because of the magnetism of the Fe_3_O_4_ NPs. The results in Figure 12b and Figure 2c indicate that **P2-5%** ES nanofibers have the potential for application in multifunctional sensory filter membrane devices for HTM ion chelation, temperature sensing, and magnetism. In conclusion, these copolymer inorganic NP-based sensory fibers have considerable potential for application in water purification sensing filters for the filtration of industrial wastewater with HTM, and may assist researchers in purifying water while simultaneously chelating and sensing Hg^2+^; moreover, used magnetic fluorescent switchable chemosensors can be collected using magnets (noncontact force).

## 4. Conclusions

Novel magnetic fluorescent switchable chemosensors for the simultaneous detection of temperature, magnetism, and Hg^2+^ based on fluorescent ES nanofibers were prepared using blends of poly(NIPAAm-*co*-NMA-*co*-AA), BNPTU, and Fe_3_O_4_ NPs by employing a single-capillary spinneret. The NIPAAm, NMA, AA, BNPTU, Fe_3_O_4_ NPs moieties were designed to provide thermoresponsiveness, chemical cross-linking, dispersion of Fe_3_O_4_ NPs, sensing of Hg^2+^, and magnetism, respectively. Cross-linked ES nanofibers maintained their structure in water and exhibited sensitivity toward temperature variations and Hg^2+^ because of the sufficient NMA composition. The fluorescence emission of BNPTU within the ES nanofibers exhibited strong selectivity toward Hg^2+^ with green emission in aqueous solutions without Hg^2+^ (thiourea-derived), shifting to blue emission in aqueous solutions with Hg^2+^ (imidazoline-derived). The **P2-5%** ES nanofibers exhibited considerable blueshifts in photoluminescence spectra and enhanced emission intensity for detecting an extremely dilute concentration of Hg^2+^ (10^−3^ M). Furthermore, the LCST of the NIPAAm moiety in the **P2-5%** nanofibers showed a significant temperature-dependent variation in PL intensity due to fiber volume change (or hydrophilic to hydrophobic change), engendering distinct on–off switching of photoluminescence when the nanofibers were exposed to Hg^2+^. Furthermore, a magnet can directly attract the **P2-5%** ES nanofibers because of the magnetism of the Fe_3_O_4_ NPs, which serves as a substitute for removal through other methods. The present study demonstrated that the prepared magnetic fluorescent ES nanofibers can be used as naked eye sensors and have potential for application in multifunctional environmental sensing devices.

## Figures and Tables

**Table 1:** Polymerization conditions and molecular weights of poly(NIPAAm-*co*-NMA-*co*-AA) random copolymers.

Polymer No.	Composition ^a^ NIPAAm:NMA:AA	*M*_n_^b^	PDI	*T*_d_ (°C)	LCST (°C) (in pH = 7)
**P1**	10:5:0	26,856	2.01	375	54.5
**P2**	10:5:3	18,041	1.76	374	58.1

**^a^** Molar ratio (%) estimated from ^1^H-NMR spectra. ^b^ Determined using THF eluent.

## Supplementary Materials

The following are available online at www.mdpi.com/2073-4360/9/4/136/s1. Figure S1: GPC profiles of **P1** and **P2** copolymers. Figure S2: TGA curves of **P1** and **P2** copolymers with a heating rate of 10 °C min^−1^ in a nitrogen atmosphere. Figure S3: Variations in optical transmittance of **P1** and **P2** in pH 7 water solutions with temperatures between 30 and 60 °C. Figure S4: TGA curves of **P2-5%** blended with 5 wt % Fe_3_O_4_ NPs nanofibers with a heating rate of 10 °C min^−1^ in a nitrogen atmosphere. Figure S5: PL intensity of pristine BNPTU compound as the temperature is increased from 30 to 60 °C. Table S1: Time-dependent solution conductivity of the prepared Hg^2+^ solution.
