# Supplementary material for: Novel Magnet and Thermoresponsive Chemosensory Electrospinning Fluorescent Nanofibers and Their Sensing Capability for Metal Ions
Source: Polymers (Basel). 2017 Apr 10;9(4):136. doi: 10.3390/polym9040136 (PMC6432252; doi:10.3390/polym9040136)
Supplement: Supplementary file 1 [file polymers-09-00136-s001.pdf]

Supplementary Materials: Novel Magnet-Responsive Chemosensory Electrospinning Fluorescent Nanofibers and Their Multifunctional Sensing Capability for pH, Temperature, and Metal Ions

Fang-Cheng Liang [1,2], Yi-Ling Luo [1], Chi-Ching Kuo [1,*], Bo-Yu Chen [1], Chia-Jung Cho [1], Fan-Jie Lin [1], Yang-Yen Yu [3,4] and Redouane Borsali [2]

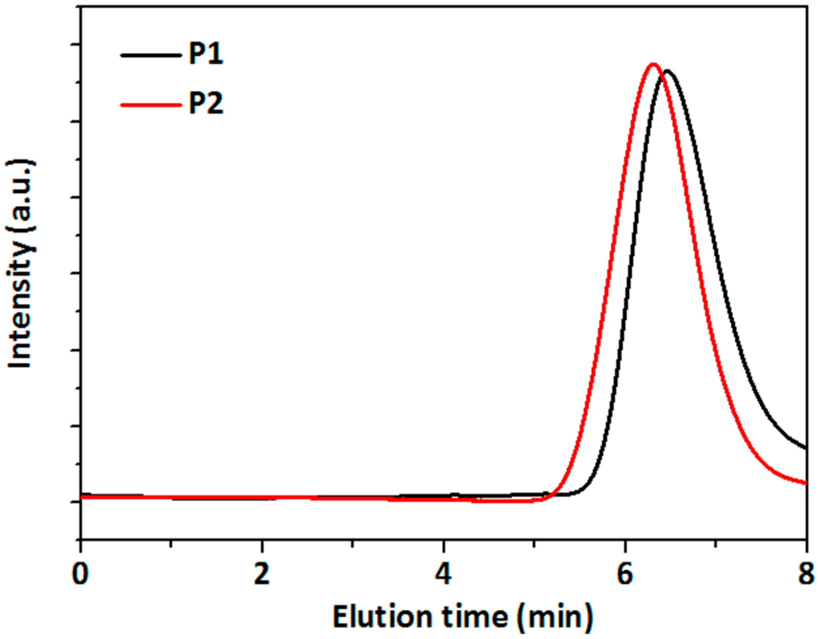

Figure S1. GPC profiles of **P1** and **P2** copolymers.

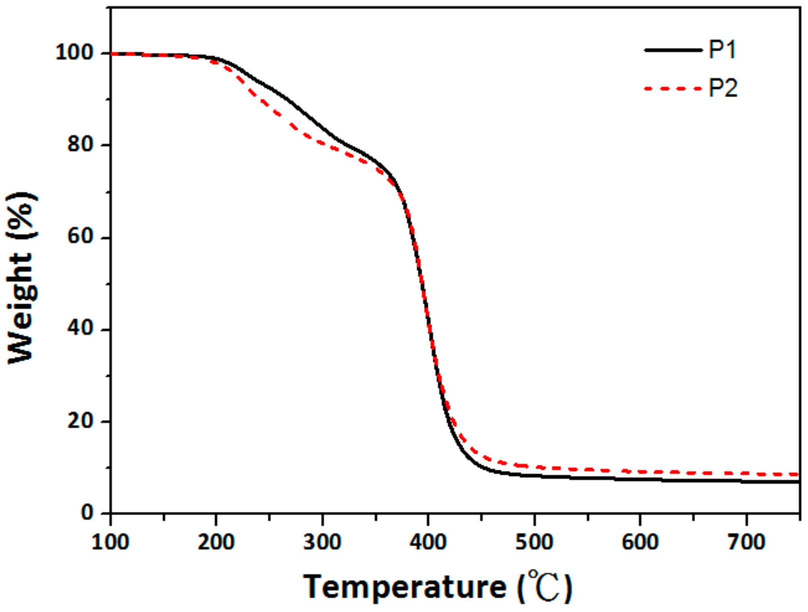

Figure S2. TGA curves of **P1** and **P2** copolymers with a heating rate of 10 °C min^{-1} in a nitrogen atmosphere.

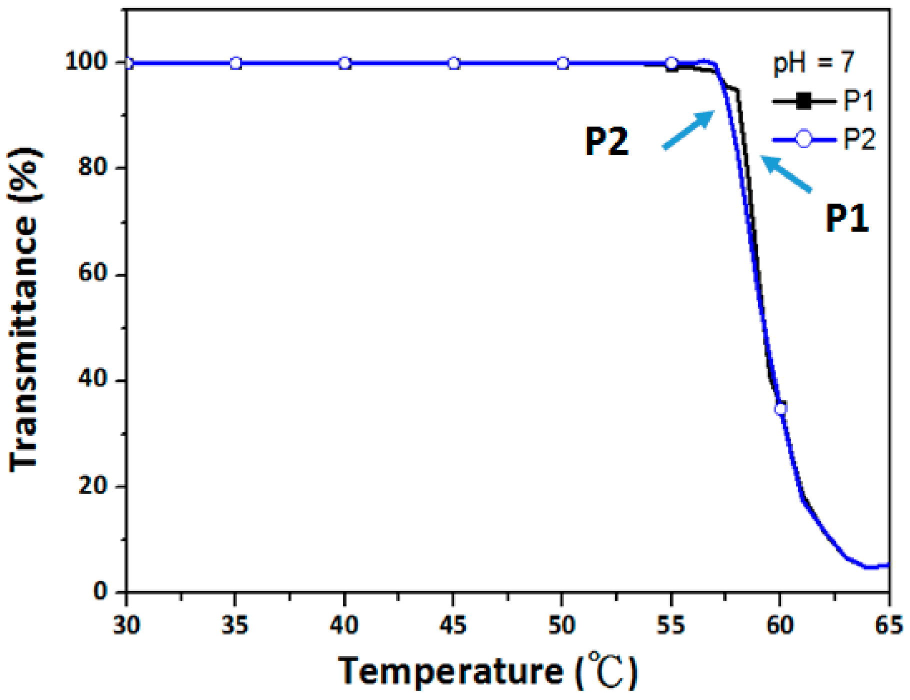

Figure S3. Variations in optical transmittance of **P1** and **P2** in pH 7 water solutions with temperatures between 30 and 65 °C.

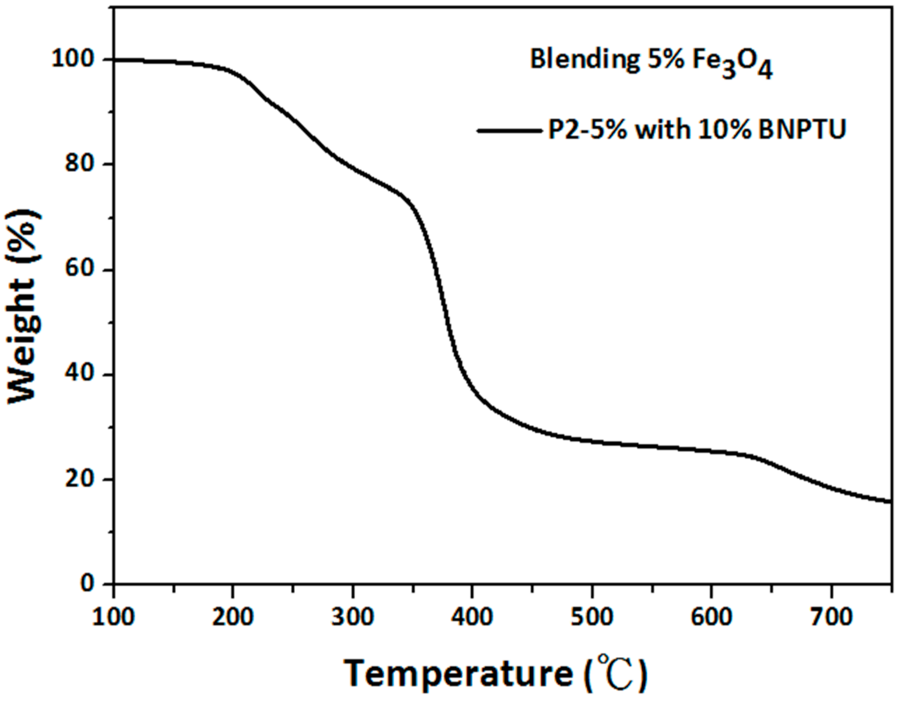

Figure S4. TGA curves of **P2-5%** blended with 5 wt% Fe_3O_4 NP nanofibers with a heating rate of 10 °C min^{-1} in a nitrogen atmosphere.

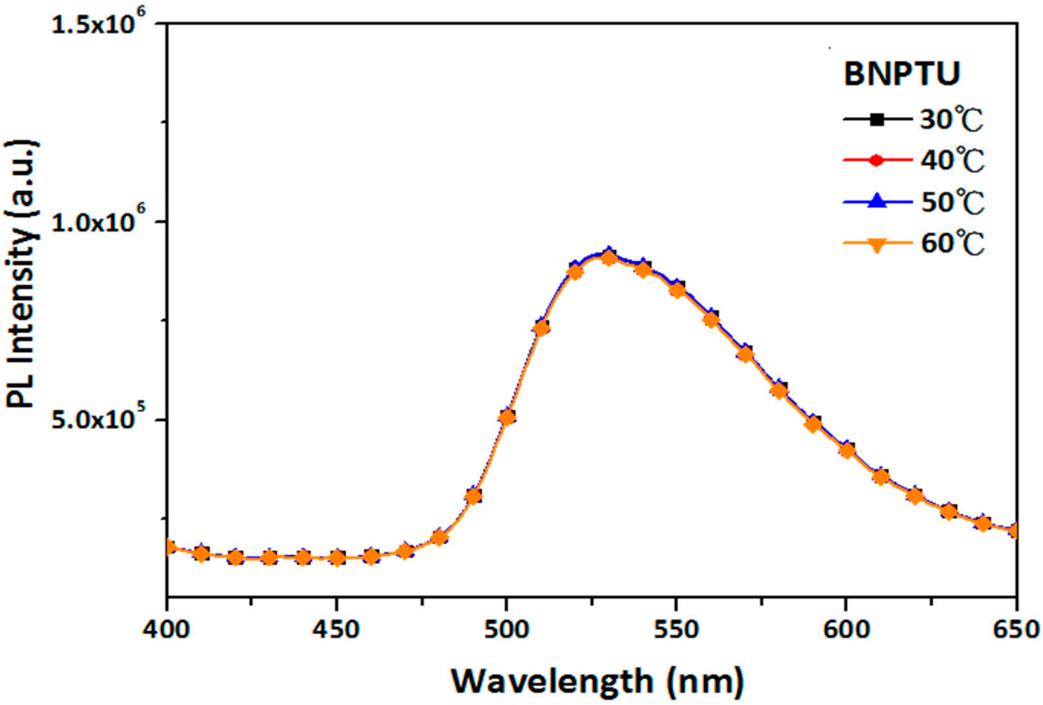

Figure S5. PL intensity of pristine BNPTU compound under a temperature increase from 30 to 60 °C.

Table S1. Time-dependent solution conductivity of the prepared Hg^{2+} solution.

Time (min)	Conductivity (μS/cm)
0	131.2
5	124.5
10	112.8
15	118.3
20	101.4
